# *Capsicum chinensis* L. growth and nutraceutical properties are enhanced by biostimulants in a long-term period: chemical and metabolomic approaches

**DOI:** 10.3389/fpls.2014.00375

**Published:** 2014-08-01

**Authors:** Andrea Ertani, Diego Pizzeghello, Ornella Francioso, Paolo Sambo, Santiago Sanchez-Cortes, Serenella Nardi

**Affiliations:** ^1^Dipartimento di Agronomia, Animali, Alimenti, Risorse Naturali e Ambiente, Università di PadovaPadova, Italy; ^2^Dipartimento di Scienze Agrarie, Università di BolognaBologna, Italy; ^3^Spanish National Research Council (CSIC)-Instituto de Estructura de la Materia (IEM)Madrid, Spain

**Keywords:** FT-IR, Raman, HRMAS-NMR, alfalfa hydrolyzed, red grape extract, flowering and maturity, phenolic acids, carbohydrates

## Abstract

Two biostimulants, one derived from alfalfa plants (AH) and the other obtained from red grape (RG), were chemically characterized using enzyme linked immuno-sorbent assays, Fourier transform infrared (FT-IR) and Raman spectroscopies. Two doses (50 and 100 mL L^−1^ for RG, and 25 and 50 mL L^−1^ for AH) of biostimulants were applied to *Capsicum chinensis* L. plants cultivated in pots inside a tunnel. The experimental design consisted of the factorial combination of treatment (no biostimulant, plus AH, plus RG) at three doses (zero, low, and high) and two time-course applications (at the second and fourth week after transplantation) and the effects were recorded at flowering and maturity. Both biostimulants contained different amounts of indoleacetic acid and isopentenyladenosine; the AH spectra exhibited amino acid functional groups in the peptidic structure, while the RG spectra showed the presence of polyphenols, such as resveratrol. These results revealed that at flowering, RG and AH increased the weights of fresh leaves and fruits and the number of green fruits, whereas at maturity, the biostimulants most affected the fresh weight and number of red fruits. At flowering, the leaves of the treated plants contained high amounts of epicatechin, ascorbic acid, quercetin, and dihydrocapsaicin. At maturity, the leaves of the treated plants exhibited elevated amounts of fructose, glucose, chlorogenic, and ferulic acids. Moreover, green fruits exhibited a high content of chlorogenic acid, *p*-hydroxybenzoic acid, *p*-coumaric acid and antioxidant activity, while both AH- and RG-treated red fruits were highly endowed in capsaicin. The ^1^H high-resolution magic-angle spinning (HRMAS)-nuclear magnetic resonance (NMR) spectra of red fruits revealed that both products induced a high amount of NADP^+^, whereas RG also increased glucose, fumarate, ascorbate, thymidine and high molecular weight species. Our results suggested that AH and RG promoted plant growth and the production of secondary metabolites, such as phenols.

## Introduction

The long-term application of excessive doses of inorganic and organic fertilizers have resulted in a dramatic increased risk of nitrate and phosphate losses to aquatic ecosystems (Pizzeghello et al., 2011; Sebilo et al., 2013), which subsequently cause pollution and a reduction in environmental health. Consequently, one interesting research focus in the field of agriculture is the study of specific bio-products, which are capable of positively influencing plant growth and, at the same, enabling the reduction of fertilizer rates. Among these bio-products include biostimulants, which have become more important due to their organic origin, which include cultivation wastes, fruit and skin processing, and their very low application doses. The 1st World Congress on the use of biostimulants in agriculture held in 2013 in Strasbourg (France) defined plant-biostimulants as “*Substances and materials, with the exception of nutrients and pesticides, which, when applied to plants, seeds or growing substrates in specific formulations, have the capacity to modify physiological processes of plants in a way that provides potential benefits to growth, development and/or stress response*” (du Jardin, 2012). The most frequent effects induced by biostimulants are an improved activity of soil microbiota (Corte et al., 2014), an increased production of growth regulators-like activity in both soil (Frankenberger and Arshad, 1995) and plants (Jindo et al., 2012; Pizzeghello et al., 2013), and an increased root development, which favors the adsorption of nutrients (Canellas et al., 2002; Nardi et al., 2002; Khan et al., 2009). In plants, biostimulants stimulated numerous metabolic pathways (Nardi et al., 2007, 2009; Schiavon et al., 2008; Ertani et al., 2012). Specifically, a cellulosolytic apple hydrolysate and a blueberry extract have been shown to increase maize growth via the induction of nitrogen and phenylpropanoid pathways, which increased photosynthetic efficiency (Ertani et al., 2011). This might favor the high accumulation of sugars in fruits, fruit sets and size (El-Nemr et al., 2012). Moreover, biostimulated crops are also less sensitive to stressful conditions (i.e., drought, extreme temperatures, excessive moisture in the rhizosphere, over- or under-exposure to light and salinity) (e.g., Marfà et al., 2009; Ertani et al., 2013) due to their high production of anti-oxidant compounds (Lakhdar et al., 2010). In any case, the peculiarity of biostimulants is that the physiological responses are not attributable to their macro and micro nutrient contents but rather to the presence of activator compounds, such as endogenous hormones, small peptides, phenolics, and triacontanol (Jindo et al., 2012; Ertani et al., 2013; Pizzeghello et al., 2013). In fact, the maximum efficiency of a biostimulant occurs at very low dosages and is dependent on plant species, cultivars and vegetative phase (Nardi et al., 2000; Zhang et al., 2003; Kaufmann et al., 2007; Ertani et al., 2011).

The majority of studies on the effects of biostimulants in plants involve short-term experiments with seed germination and young plantlet growth, and little information is available on the maturity phase (Nardi et al., 2002, 2009; Rose et al., 2014). Recently, Pascual et al. (2008) found that the application of sewage sludge positively affected the growth and fruit yield of pepper plants. Such effects were associated with an increased nutrient availability in the substrate, as well as an improvement in microbial activity. In contrast, the same group of authors (Azcona et al., 2011) reported that humic substances (HSs) from composted sewage sludge increased dry-matter production and leaf area at early stages of pepper development, whereas fewer differences were observed at maturity.

A more detailed study of the effects induced by biostimulants during an entire cycle of culture was performed in which pepper plantlets were supplied with biostimulants, and the effects on plant growth, fruit yield and chemical composition were recorded at two different development phases (flowering and maturity). Pepper was selected because it is a representative species of the Mediterranean region and it has a short developmental period. In addition, it also has a long history as a source of healthy and biologically active compounds, including flavonoids, phenols, carotenoids, capsaicinoids, vitamins, and anticancer properties (De Masi et al., 2007). For this study, we employed two different biostimulant-products, in which one of the products exhibits high bioactivity (Schiavon et al., 2008; Ertani et al., 2009, 2013). The biostimulants were chemically characterized using vibrational spectroscopy (Fourier transform infrared, FT-IR and Raman spectroscopies), and the metabolite changes induced in the red fruits of the biostimulated-plants were estimated using high-resolution magic-angle spinning (HRMAS)-nuclear magnetic resonance (NMR). Metabolomics examines the most abundant low molecular weight compounds, i.e., the metabolome, that are present in any biological matrix; metabolomics is defined as the systematic study of the unique chemical fingerprints that specific cellular processes leave behind. HRMAS-NMR spectroscopy has recently been proposed as a reliable tool to assess the metabolomes of food products and offers the unique opportunity of measuring samples in the absence of any chemical and/or physical preparation by producing highly resolved NMR spectra.

## Materials and methods

### Chemical and spectroscopic characterization of biostimulants

Two biostimulants manufactured by ILSA S.p.A. (Arzignano, VI, Italy) were used. One was produced by fully controlled enzymatic hydrolysis using alfalfa (*Medicago sativa* L.) plants (alfalfa hydrolyzed, AH), and the second was obtained by cool extraction (Machado, 2007) of red grape skin material of *Vitis vinifera* L. and hereinafter will be called RG.

The chemical and physical properties of AH have been reported elsewhere (Schiavon et al., 2008; Ertani et al., 2009). For RG, the pH was determined in water (3:50 w/v) (Trinchera et al., 2003) and total organic carbon (TOC) using an element analyser (varioMACRO CNS, Hanau, Germany). Total phenols and sugars were determined according to Arnaldos et al. (2001) and Ertani et al. (2011), respectively. The content of two hormones (indoleacetic acid, IAA and isopentenyladenosine, IPA) was quantified using enzyme linked immuno-sorbent assays (ELISA) (Sigma, St. Louis, MO, USA) as previously described in Pizzeghello et al. (2013).

Infrared spectral acquisition was performed on solid samples using a Nicolet 5700 FT-IR equipped with a diamond attenuated total reflectance (ATR) accessory and a DTGS (Nicolet, Madison, USA) detector. The total number of scans averaged for each spectrum was 64 with a resolution of 4 cm^−1^. The background spectrum was acquired in air. Spectra analysis was performed with Grams/386 spectral software (Galactic Industrious Corp., Salem, NH, USA).

Raman spectra of samples were registered in solid state with a Renishaw Raman RM2000 instrument, which was equipped with an electrically cooled CCD camera and an excitation line using a He/Ne laser at 632.8 nm. Each spectrum was registered using a 10 s measurement time.

### Experimental design and plant growth

The experimental trial was derived from the factorial combination of three types of treatments, no treated (UNT), a cool extract from red grape (RG), and an alfalfa hydrolyzed (AH), with two application doses, which were supplied at two randomized blocks of pots, consisting of ten replications for a total of 100 pots. The pots were filled with 2 L perlite/vermiculite (1/1 w/w) mixture per pot. At the start of the trial, 7 day-old pepper seedlings (*Capsicum chinense* L. cv. Fuoco della Prateria) were homogenously selected per growth and one seedling was transplanted per pot. The plants were grown until maturity in a tunnel maintained at 25/15°C day/night, receiving natural light. Treatment was applied by spraying the diluted products on the surface of the leaves at two dosages: 50 and 100 mL L^−1^ for RG, and 25 and 50 mL L^−1^ for AH by spraying each one with 4.5 mL of RG or AH on the leaves. The plants were treated at the second and fourth week after transplantation, and two collection periods were used: at flowering (time 1) and maturity (time 2) (4- and 6-week-old plants after transplantation, respectively). All treatments were irrigated with half-strength Hoagland's nutrient solution (Hoagland and Arnon, 1950) to eliminate nutrient limitation. The plants were randomly collected, and the leaves were separated from the fruits, weighed and washed in deionized water. Sub-samples were immediately frozen with liquid nitrogen and kept at −80°C for further chemical analyses.

### Plant metabolic compounds

#### Chemical determination of metabolic compounds

Total phenols (TPs) were determined according to Arnaldos et al. (2001) with gallic acid used as a standard (Sigma), and total sugars were determined as previously described by Nicoletto et al. (2013). The oxygen radical absorbance capacity (ORAC) assay was performed according to the method of Madhujith and Shahidi (2009), using a FLUOstar OPTIMA microplate reader (BMG Labtechnologies GmbH, Offenberg, Germany) equipped with FLUOstar OPTIMA evaluation software version 1.30- 0 and black polystyrene, non-treated 96-well microplates (Costar Corning Inc., Corning, NY).

Ascorbic acid was extracted from five grams of tissue, homogenized until uniform consistency in a meta-phosphoric acid and acetic acid solution. The concentration of ascorbic acid was determined according to the ISO 6557 method.

To determine the levels of capsaicin and dihydrocapsaicin, two grams of tissue was extracted by treatment with 20 mL of acetone and homogenization with an Ultra-Turrax T 25 (IKA, Germany) for 30 s at 17,500 rpm. The extracts were filtered through a Whatman No. 42 filter paper followed by a 0.45-μm nylon membrane prior to high-performance liquid chromatography (HPLC) analysis. A total of 10 extracts were analyzed. The HPLC system consisted of a model (X-LC Jasco Co., Japan) equipped with a DAD detector (MD- 2015, Jasco Co., Japan) and autosampler (AS-2055 Jasco Co., Japan). A ODS-2 (250 × 45 mm, 5 μm Tracer Extrasil) column was eluted with methanol/H_2_O (50:50) at a flow rate of 1.0 mL min^−1^ at 25°C; the detection was performed at 278 nm; the separation was obtained in isocratic elution for 10 min and then a linear gradient of 50–90% methanol (Sigma) for 10 min. The capsaicin and dihydrocapsaicin concentration in the extracts was obtained using a stock solution of standard capsaicin (2 mg L^−1^) (Fluka, St. Louis, MO, USA) and dihydrocapsaicin (2 mg L^−1^) (Fluka) in methanol (Sigma), transferred into a vial and kept cool at 4°C prior to use.

β-Carotene and lycopene were extracted and quantified using ultraviolet-visible (UV Vis) spectrophotometric assays as previously described by Rodríguez-Amaya (2001). These results are expressed in μg g^−1^ dry weight.

#### NMR measurements in red peppers

The samples were prepared by inserting ca. 3–5 mg of lyophilized red pepper in a 4-mm HRMAS rotor with a 50 μL spherical insert. Approximately 40 μL of 0.1 M D_2_O phosphate buffer (pH 7.2) with 0.5% TSP, i.e., 3-(trimethylsilyl)-propionic- 2,2,3,3-d4 acid sodium salt, were then added. The HRMAS-NMR spectra were recorded at 298 K using a Bruker AVANCE spectrometer operating at a ^1^H frequency of 400.13 MHz, equipped with a 4 mm HRMAS dual channel probe head and spinning samples at 7 kHz. ^1^H NMR spectra were referenced to the methyl group signal at δ 0.00 ppm of TSP, while the ^13^CNMR spectra were referenced to the TSP δ 0.00 ppm. The ^1^H-HRMAS-NMR spectra were acquired using a water suppression pulse sequence, noesypr1D (Bruker library), using 32 K data points over a 4807 Hz spectral width and addition of 256 transients. A recycle delay of 3 s and a delay for the efficient NOE effect equal to 150 ms were used. The 90° pulse length was 5.3 μs, and saturation of the water residual signal was achieved by irradiating during recycle delay at δ equal to 4.70 ppm. Each spectrum was FT transformed with 64 K data points and manually phased and base-lined, and a line broadening factor equal to 0.3 Hz was applied to the FID prior FT. ^13^C-HRMAS-NMR spectra were acquired using the power-gated decoupling sequence, zgpg30 (Bruker library), with a 30° flip angle pulse of 5.0 μs. The experiments were performed using 64 K data points over a 22,123 Hz (220 ppm) spectral width by the addition of 64 K transients with a recycle delay of 3 s. Each spectrum was FT transformed with 128 K data points and manually phased and base-lined, and a line broadening factor of 0.5 Hz was applied to the FID. The ^1^H-^1^H TOCSY experiment was acquired in the TPPI phase sensitive mode, with a 4807 Hz spectral width in both dimensions, 100 ms of spin-lock time of 4500 Hz, 2 K data points in f2, and 1 K increments in f1, each with 32 scans. The ^1^H-^13^C HSQC spectra were acquired in the TPPI phase-sensitive mode, with a 4807 Hz spectral width in f2 dimension and a 15,083 Hz spectral width in f1. 2 K data points in f2 and 1 K increments in f1, each with 32 scans, were used. All of the ^1^H NMR spectra were baseline corrected, and aligned using XWINNMR 3.5 software (Bruker Biospin, Karlsruhe, Germany). Each spectrum was divided into intervals equal to 0.06 ppm (buckets) in a range from 0.06 to 9.00 ppm, with the exclusion of the water region from 4.74 to 4.86 ppm, using AMIX 3.5 software (Bruker Biospin, Karlsruhe, Germany). All integrated buckets were scaled by using the ACD bucketing method within ACDlab 8.0 software to a signal intensity of the peak at 3.81 ppm such that the NMR spectra were bucketed in 149 variables. The area under each bin was integrated and normalized with respect to the sum of all integrals, which was set equal to 100. The NMR data were analyzed using the means of the partial least squares projections to latent structures–discriminant analysis (PLS-DA) as previously described in Ritota et al. (2013) and Pacifico et al. (2013).

### Statistical analysis

Bartlett's test was performed on the data to test the homogeneity of variance. A multiple-way completely randomized ANOVA was used to compare treatment effects. The factors considered were treatment (UNT, RG and AH), concentration (0, low and high dosage), time from treatment (flowering and maturity), and maturation stage of fruits (red and green peppers). To identify the structure of the interdependences of the main parameters of the leaves and fruits, joint principal components analysis (PCA) was performed on the following variables: the weight of fresh leaves and fruits, glucose, fructose, total phenolic acids, chlorogenic, caffeic, ferulic, *p*-coumaric, *p*-hydroxybenzoic, cinnamic, and epicatechic acids, quercetin, ß-carotene, capsaicin, dihydrocapsaicin, lycopene, ascorbic acid, and antioxidant activity. The standardized variables were subjected to PCA; and the rotated orthogonal components (varimax rotation method) were extracted and the relative scores were determined. Only PCs with an eigenvalue >1 were considered for discussion. Differences between groups of means were obtained using the Student-Newman-Keuls test at *P* ≤ 0.05. All statistics were made by SPSS software version 19 (SPSS inc., 1999).

## Results

### Chemical and spectroscopic features of biostimulants

The chemical characteristics of the two biostimulants are listed in Table 1. AH strongly differed from RG for pH value (5.9 and 2.9, respectively) (*P* ≤ 0.05), TOC (18.8 and 1.23 %) (*P* ≤ 0.05), total phenols (2576 and 970 mg L^−1^) (*P* ≤ 0.05), and IAA (18.46 and 2.92 nmol mg^−1^ C) (*P* ≤ 0.05). Smaller differences were found in the amount of total sugars (*P* ≤ 0.05) and IPA, which were slightly higher in RG (5700 mg L^−1^ and 0.073 nmol mg^−1^ C, respectively) compared to AH (4642 mg L^−1^ and 0.055 nmol mg^−1^ C, respectively).

**Table 1:** **Chemical properties and content in the hormones of red grape (RG) and alfalfa hydrolyzed (AH) biostimulants (*n* = 5; ± standard deviation)**.

**Property**	**Unit**	**RG**	**AH**
[H^+^]	pH	2.9±0.13	5.9±0.28
TOC	%	1.23±0.06	18.8±0.90
Total sugars	mg L^−1^	5700±210	4642±151
Total phenols	mg L^−1^	970±45	2576±110
IAA	nmol mg^−1^ Carbon	2.92±0.12	18.46±0.85
IPA	nmol mg^−1^ Carbon	0.073±0.010	0.055±0.008

*IAA, indoleacetic acid; IPA, isopentenyladenosine; TOC, total organic carbon*.

**Figure 1:**
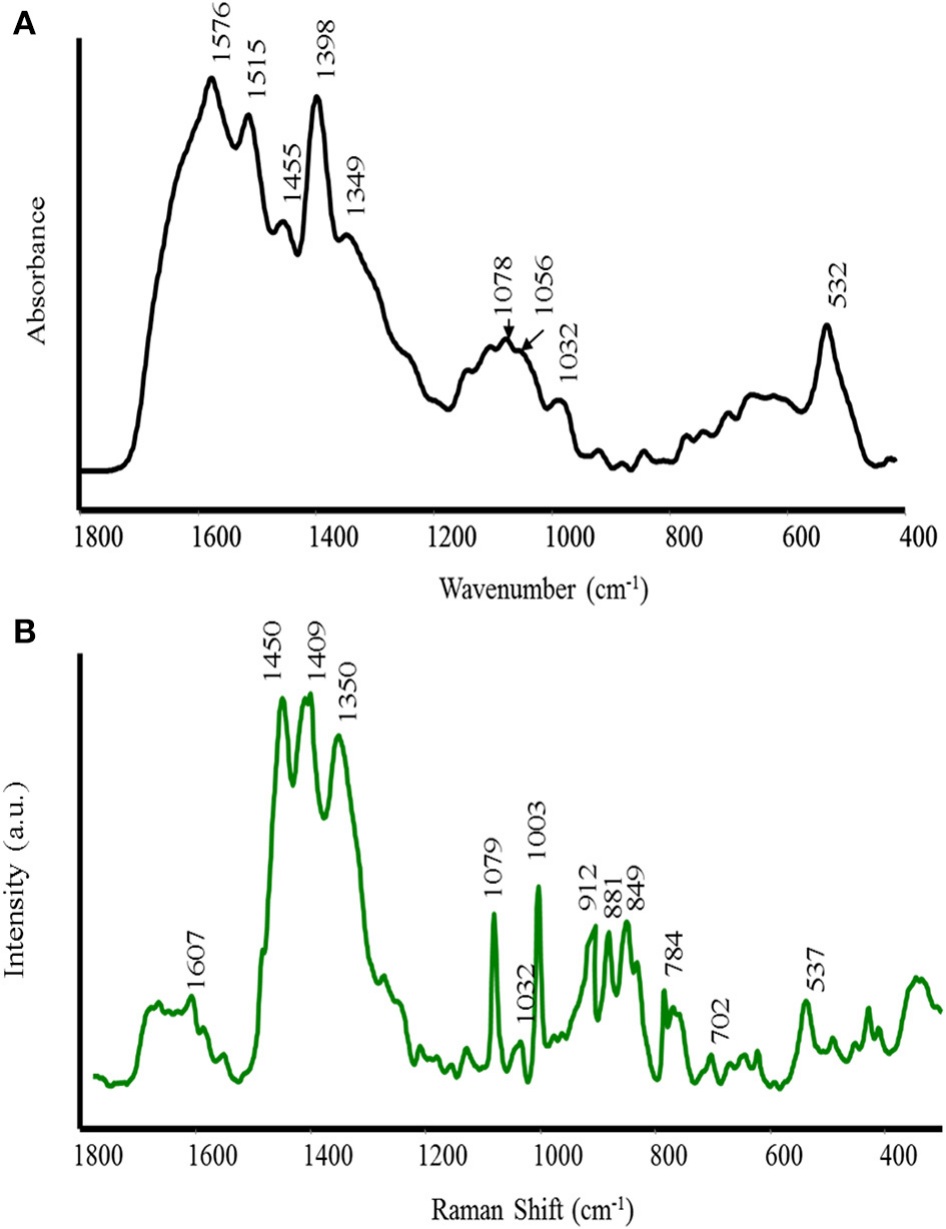
**FT-IR (A) and Raman (B) spectra of alfalfa hydrolyzed (AH) biostimulant**.

The FT-IR and Raman spectra of AH are shown in Figure 1 (A IR and B Raman). The main attributions of the bands were performed according to the literature (Barth, 2000; Culka et al., 2010). Our interest was focused on the 1800–400 cm^−1^ spectral region because in this area, the characteristic groups are absorbed and the “finger fingerprint” region is included. Consequently, in this region, any structural differences can be detected. Both spectra are characterized by a number of complementary bands of varying intensities. The bending vibration of the NH_2_ group appears as a shoulder at 1630 cm^−1^ in the FT-IR spectrum. Similarly, the NH_2_ bending vibration in the Raman spectrum is very weak and is located at 1607 cm^−1^. In the IR spectrum, the strong band at 1576 cm^−1^, which is absent in the Raman spectrum, is assigned to the asymmetric stretching vibration of COO^−^ in the Asp, Glu and NH_2_ bending vibration in Gln and Lys, respectively. A band at 1515 cm^−1^ is only observed in the IR spectrum and may be assigned to the stretching vibration of the aromatic ring and NH bending motion in Try and Trp. The band at 1455 cm^−1^ in the IR spectrum is assigned to the asymmetric bending of CH_3_. The corresponding band in the Raman spectrum appears at 1450 cm^−1^. The strong band at 1398 cm^−1^ and a shoulder at 1349 cm^−1^ in the IR spectrum are attributed to the symmetric stretching vibration of COO^−^ and symmetric bending of CH_3_, respectively. The corresponding bands in the Raman spectrum appear at 1409 and 1350 cm^−1^. A medium band in the IR spectrum at 1078 cm^−1^ can also be attributed to the ρ (NH_2_) and C-O stretching vibration. The bands at 1203, 1110–1100, 1068–1012 cm^−1^ correspond to the stretching vibration of C–O instead the bands in the range 996–665 cm^−1^, which are assigned to aromatic CH out-of-plane bending vibrations.

**Figure 2:**
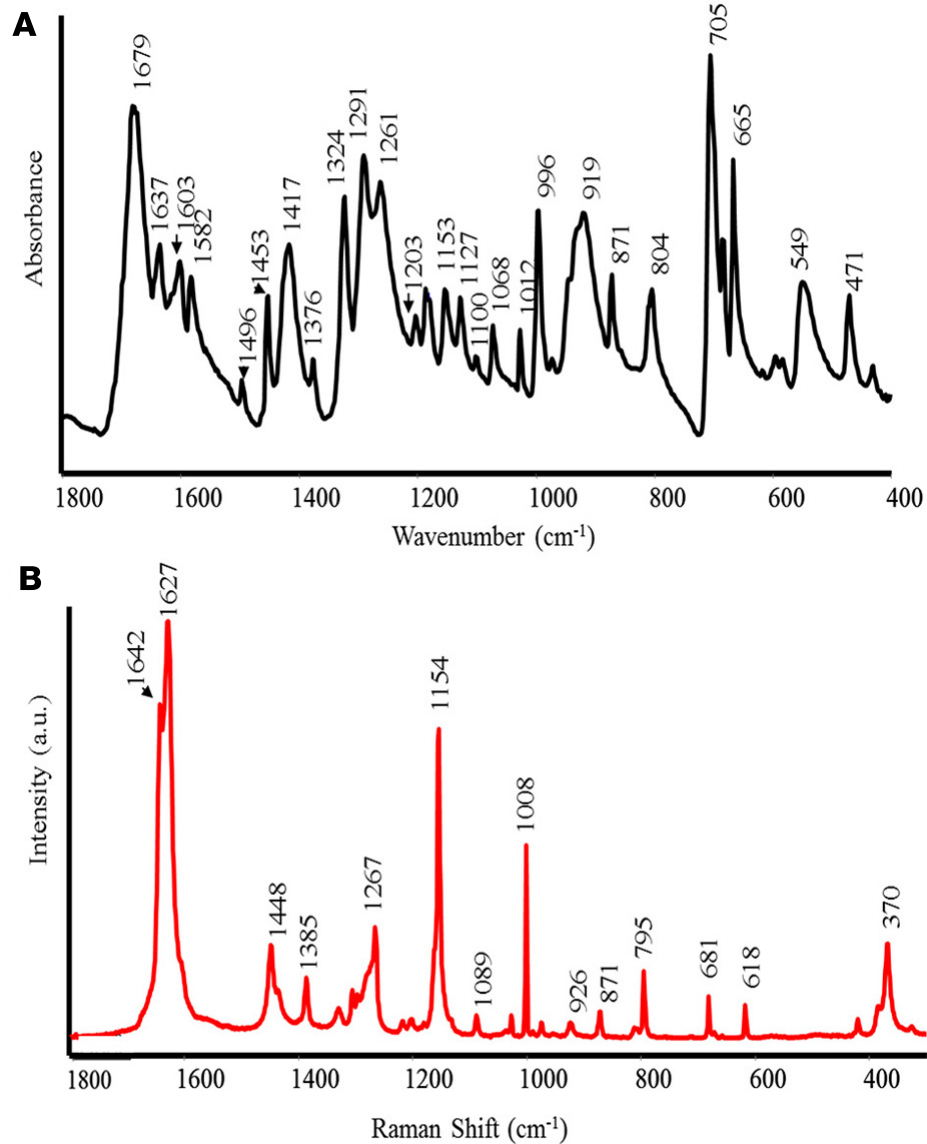
**FT-IR (A) and Raman (B) spectra of red grape (RG) biostimulant**.

The FT-IR and Raman spectra of RG are shown in Figure 2 (A IR and B Raman). The peak at 1679 cm^−1^, in the FT-IR spectrum, is assigned to the stretching vibration of carbonyl (C=O) in the aldehyde group (Bellamy, 1975). The bands at 1637, 1603, 1582, and 1496 cm^−1^ correspond to the stretching of aromatic C=C (Nakanishi and Solomon, 1977). Consequently, the peak at 1637 cm^−1^ might indicate the presence of aromatic rings in anthocyanins (Merlin et al., 1994). The peaks of moderate intensity in the range at 1453–1376 cm^−1^ correspond to the bending of −CH_3_ and −CH_2_ vibrations. Furthermore, in the same spectral region, the phenyl nucleus (C=C) absorbs (Bellamy, 1975). The peak at 1291–1261 cm^−1^ corresponds to the in-plane bending of O–H (Bellamy, 1975) and stretching vibration of C–O in phenols. The bands at 1203, 1110–1100, 1068–1012 cm^−1^ correspond to the stretching vibration of C–O instead of the bands in the range 996–665 cm^−1^, which can be assigned to aromatic CH out-of-plane bending vibrations.

The Raman spectrum shows intense peaks without large fluorescence emission. This spectrum exhibits two intense bands at 1642 and 1627 cm^−1^, which correspond to ν (C=C), indicating the existence of both aliphatic and aromatic C=C double bonds as in the case of the polyphenols caffeic and isoferulic acids (Sanchez-Cortes and Garcia-Ramos, 2000). The bands at 1448 and 1436 cm^−1^ are assigned to δ (CH_2_) and δ (CH_3_), although some contribution from ring stretching and δ (OH) is also possible in these bands. The medium band appearing at 1267 cm^−1^ is attributed to the ν (C–O), characteristic of phenolic compounds as also detected in the FT-IR spectrum. The very intense band at 1154 cm^−1^ is assigned to δ (CH) in aromatic compounds, but this band is also characteristic of polyenes, such as carotenoids and in the polyphenol resveratrol (Billes et al., 2007). The latter band may be coupled to ν (C–C) vibrations in aromatic compounds linked to aliphatic C=C double bonds. The weak and medium bands appearing in the 1100–900 cm^−1^ region are attributed to δ (CH) of the aromatic moieties and ν (C-C) of the aliphatic parts. The bands observed at 900–600 cm^−1^ can be attributed to skeletal vibrations, i.e., vibrations involving δ (CCC) motions. Finally, the strong band appearing at 370 cm^−1^ is attributed to bending vibrations in polyphenols δ (CCO) and also to δ (CC=C) bending of the vinylidene group in resveratrol (Billes et al., 2007). The high similarity between the Raman spectrum of the grape skin and resveratrol indicates that most of the bands observed in the latter sample might be due to the presence of a large amount of this polyphenol in the sample.

### Weight and number of fresh leaves and fruits

ANOVA analysis revealed that the weight of fresh leaves and fruits and fruit number were affected by treatment (*P* ≤ 0.001), concentration (*P* ≤ 0.001) and time from treatment (*P* ≤ 0.001), whereas the *post-hoc* test showed significant differences between the treated and control plants (Table 2). After the first application (time 1) with AH and RG, the weight of fresh leaves, total fruit fresh weight and fresh fruit number were strongly enhanced with respect to UNT (Table 2). AH at the dose of 50 mL L^−1^, induced the highest weight of fresh leaves (2.6-fold UNT), total fruits (2.2-fold UNT) and number of fruits (2.4-fold UNT) (Table 2). Intermediate values between AH (50 mL L^−1^) and UNT were observed for RG at a rate of 50 mL L^−1^ (Table 2). Among the fruits, the most affected fruits were the green fruits, with 50 mL L^−1^ AH recorded as the highest fresh weight and number reaching values pair at 2.8-fold UNT (Table 2). Furthermore, 25 mL L^−1^ AH and 100 mL L^−1^ RG also affected the weight of fresh leaves, number of green fruits and total number of fruits, but with minor increases with respect to the previous dosages (Table 2). After the second treatment (time 2), both AH and RG induced higher values in the weight of total fruits and number of total fruits compared to untreated plants (Table 2). Among the fruits that were the most affected were the red fruits (Table 2). In particular, the fresh weight of red fruits was higher in 50 mL L^−1^ AH and 50 mL L^−1^ RG (32.7 and 31.8 g, respectively) compared to 25 mL L^−1^ AH (29.9 g) and 100 mL L^−1^ RG (23.1 g) and UNT (16.4 g) (Table 2).

**Table 2:** **Fresh weights and number of leaves and fruits (g fw, n) after the 1st and 2nd (T1 and 2) application of biostimulants (untreated, UNT; red grape, RG; alfalfa hydrolyzed, AH)**.

				**FRUITS**							
**T**	**Tr**	**C**	**LEAVES**	**G**	**O**	**R**	**TT**	**G**	**O**	**R**	**TT**
		**mL L^−1^**	**g fw**					**n**			
1	UNT	0	10.1d	14.3d	2.4b	4.6a	21.4d	18d	2	4	23d
1	RG	50	16.8b	28.7b	2.3b	2.7c	33.7b	27b	2	3	42b
1	RG	100	12.7c	19.7c	2.8a	3.0c	25.6c	21c	3	4	28c
1	AH	25	12.3c	18.6c	2.2b	3.4b	24.2c	21c	2	4	28c
1	AH	50	26.7a	40.1a	2.8a	3.4b	46.3a	50a	3	4	55a
2	UNT	0	15.4*b*	4.8*b*	0.7*c*	16.4*d*	22.0*c*	4	1*b*	12*c*	24*c*
2	RG	50	18.6*a*	3.0*e*	3.9*a*	31.8*a*	38.7*a*	5	4*a*	32*b*	36*b*
2	RG	100	14.3*b*	4.3*c*	3.3*a*	23.1*c*	30.7*b*	5	5*a*	29*b*	35*b*
2	AH	25	18.6*a*	5.3*a*	0.9*c*	29.9*b*	36.1*a*	5	2*b*	37*a*	44*a*
2	AH	50	17.4*a*	3.7*d*	2.4*b*	32.7*a*	38.8*a*	4	2*b*	37*a*	41*a*

*T, time; Tr, treatment; C, concentration; G, green; O, orange; R, red; TT, total. In the same column differences among means at T1 and T2 (italicized letters) were at P ≤ 0.05 using the Student-Newman-Keuls test*.

### Chemical composition of leaves and fruits

The chemical composition of leaves and fruits was significantly affected by treatment (*P* ≤ 0.001), concentration (*P* ≤ 0.001), time from treatment (*P* ≤ 0.001), and maturation of fruits (*P* ≤ 0.001). Significant differences between treated and untreated plants were obtained using the *post-hoc* test.

**Figure 3:**
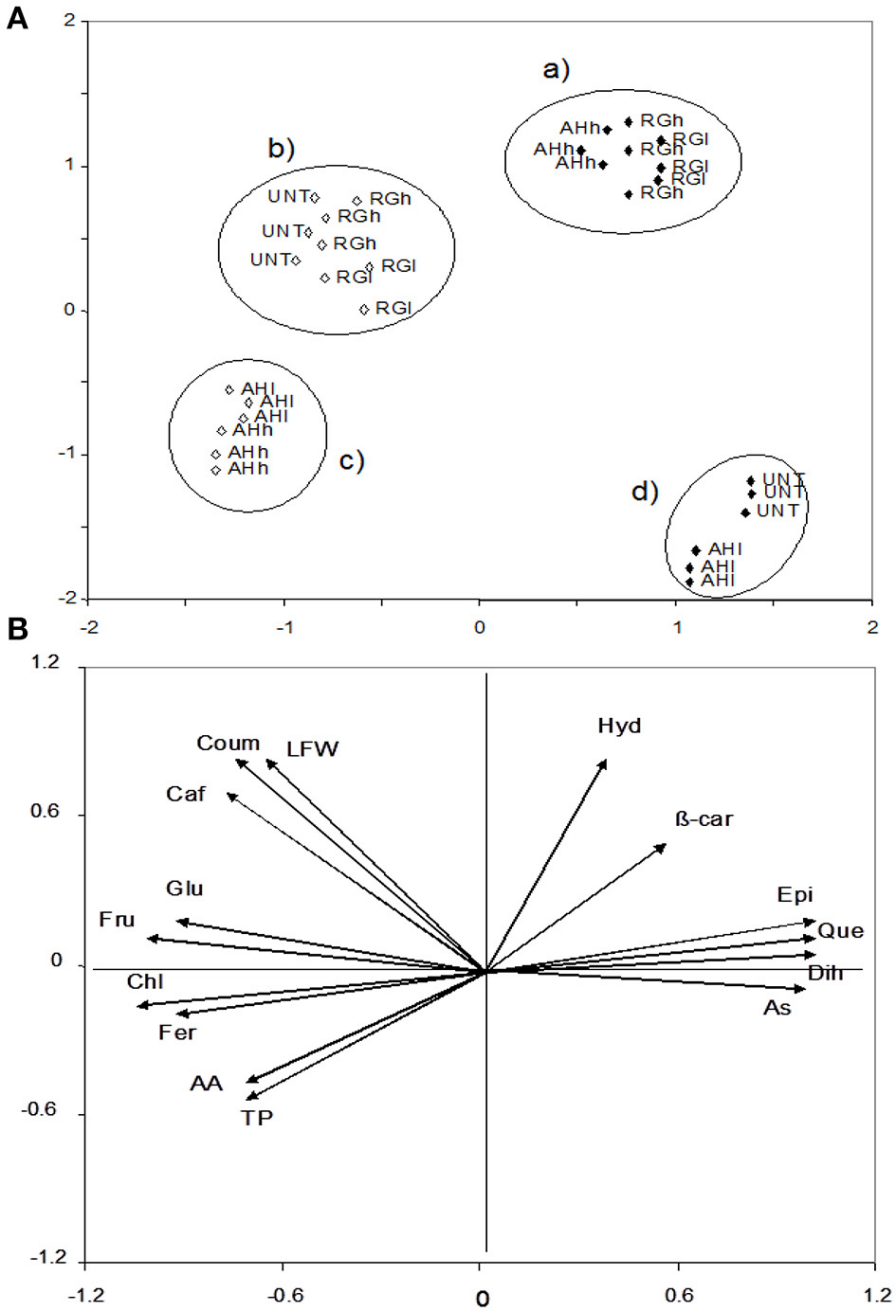
**PCA leaves scatterplot of the plants treated with and without biostimulants (upper, A) and position of the variables projected in the plane as determined by the first two principal axes (lower, B) (85% explained variance)**. Alfalfa hydrolyzed (AH) and red grape (RG) biostimulants applied at low (l) and high (h) doses, whereas no application was performed on the control (untreated, UNT). Black and white diamonds correspond to the leaves chemical composition after the 1st and 2nd application. AA, antioxidant activity; As, ascorbic acid; β-car, β-carotene; Caf, caffeic acid; Chl, chlorogenic acid; Coum, *p*-coumaric acid; Epi, epicatechic acid; Fer, ferulic acid; Fru, fructose; Glu, glucose; Hyd, *p*-hydroxybenzoic acid; LFW, leaves fresh weight; Que, quercetin; and TP, total phenols.

For the chemical characteristics of the leaves, three factors were PCA-extracted, which accounted for 96% of the variance. Factor 1 accounted for 65% of the variance and was highly correlated with dihydrocapsaicin (0.96), quercetin (0.96), ascorbic acid (0.93), and epicatechic acid (0.93), and chlorogenic acid (−0.97), fructose (−0.97), glucose (−0.90), and ferulic acid (−0.86). Factor 2 accounted for 20% and was correlated with *p*-hydroxybenzoic acid (0.85), fresh weight (0.76) and *p*-coumaric acid (0.75). The total phenolic acids (0.60) and ß-carotene (0.60) were potentially correlated with factor 3 (11% of the variance). The data were plotted according to PC1 and PC2 (Figures 3A,B), which identified four clusters corresponding to (a) first application of a high AH rate and low and high RG rates, (b) second application of low and high RG and UNT2 rates, (c) second application of low and high AH rates, and (d) first application low AH and UNT1 rates. Importantly, the first and second treatments scattered to the right and left side from the origin, indicating that time 1 (flowering) had higher values in epicatechic acid (8.14 and 2.02 μg g^−1^) (*P* ≤ 0.05), ascorbic acid (1928 and 608 mg kg^−1^) (*P* ≤ 0.05), quercetin (0.49 μg g^−1^ vs. nd) and dihydrocapsaicin (1.47 mg Fe ^2+^ kg^−1^ vs. nd) compared to time 2 (maturity) (Table 1, Supplementary Material). In contrast, elevated amounts in fructose (321 and 192 mg g^−1^) (*P* ≤ 0.05), glucose (611 and 349 mg g^−1^) (*P* ≤ 0.05), and chlorogenic (55 μg g^−1^ vs. nd) and ferulic (7.52 and 2.32 μg g^−1^) (*P* ≤ 0.05) acids were found at time 2 with respect to time 1 (Table 1, Supplementary Material). Interestingly, cluster c had higher total phenolic acids (1902 and 1583 mg GAE kg^−1^) (*P* ≤ 0.05) and antioxidant activity (3942 and 3668 mg Fe2^+^ kg^−1^) (*P* ≤ 0.05) than cluster b (Table 1, Supplementary Material). In addition, cluster d had lower values than cluster a in *p*-hydroxybenzoic acid (0.57 and 1.22 μg g^−1^) (*P* ≤ 0.05) and *ß*-carotene (1.85 and 2.27 μg g^−1^) (*P* ≤ 0.05) (Table 1, Supplementary Material). However, cluster d had higher amounts in epicatechic acid (6.94 and 2.21 μg g^−1^) (*P* ≤ 0.05), quercetin (0.42 vs. nd), dihydrocapsaicin (0.84 vs. nd), ascorbic acid (2026 and 3942 μg g^−1^) (*P* ≤ 0.05), and lower content in glucose (308 and 579 mg g^−1^) (*P* ≤ 0.05), fructose (157 and 337 mg g^−1^) (*P* ≤ 0.05), chlorogenic acid (nd vs. 66), and ferulic acid (1.27 and 11.7 μg g^−1^) (*P* ≤ 0.05) than cluster c (Table 1, Supplementary Material). Furthermore, clusters d and b differed in the higher content of *p*-coumaric acid (0.59 and 3.21 μg g^−1^) (*P* ≤ 0.05) and caffeic acid (1.54 and 5.76 μg g^−1^) (*P* ≤ 0.05) in the former compared to the latter (Table 1, Supplementary Material).

**Figure 4:**
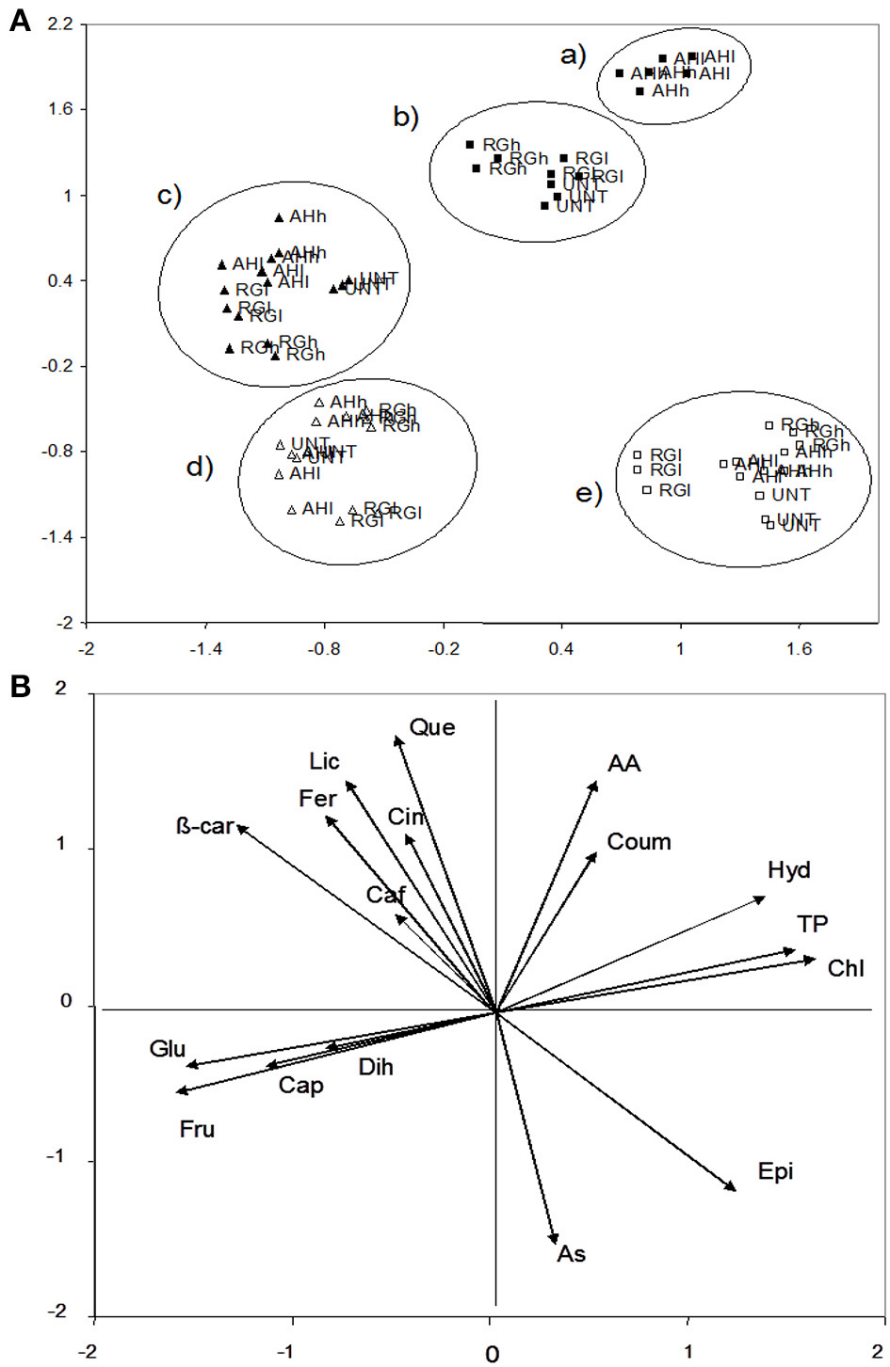
**PCA fruits scatterplot of the plants with and without biostimulants (upper, A) and the position of the variables projected in the plane as determined by the first two principal axes (lower, B) (85% explained variance)**. Alfalfa hydrolyzed (AH) and red grape (RG) biostimulants were at low (l) and high (h) doses, whereas no application was performed on the control (untreated, UNT). White and black symbols correspond to the 1st and 2nd application whereas the full square and triangle corresponded to the green and red fruits. AA, antioxidant activity; As, ascorbic acid; β-car, β-carotene; Caf, caffeic acid; Cap, capsaicin; Chl, chlorogenic acid; Cin, cinnamic acid; Coum, *p*-coumaric acid; Dih, dihydrocapsaicin; Epi, epicatechic acid; Fer, ferulic acid; Fru, fructose; Glu, glucose; Hyd, *p*-hydroxybenzoic acid; Lyc, lycopene; Que, quercetin; TP, total phenols.

For the fruit chemical characteristics, PCA extracted three factors that accounted for 80% of the variance. Factor 1 accounted for 33% of the variance and was positively correlated with chlorogenic acid (0.92), total phenolic acids (0.86), *p*-hydroxybenzoic acid (0.70), and epicatechic acid (0.67), and it was negatively correlated with fructose (−0.89), glucose (−0.85), and ß-carotene (−0.72). Factor 2 accounted for 32% and was positively correlated with quercetin (0.91), antioxidant activity (0.87), and lycopene (0.82), and it was negatively with ascorbic acid (−0.82). Caffeic acid (0.81) and dihydrocapsaicin (0.67) were potentially correlated with factor 3 (15% of the variance). The data were plotted according to PC1 and PC2 (Figures 4A,B), which identified five main clusters with axis one differing in the green fruits from the red fruits, and axis two, which could distinguish between time 1 (flowering) and time 2 (maturity). Moreover, the green fruits had a higher chlorogenic acid (20 and 2 μg g^−1^) (*P* ≤ 0.05) and total phenolic acids (4529 and 1401 mg GAE kg^−1^) (*P* ≤ 0.05) than the red fruits, whereas the red fruits were highly endowed in fructose (1903 and 508 mg g^−1^) (*P* ≤ 0.05) and glucose (1815 and 793 mg g^−1^) (*P* ≤ 0.05) with respect to the green fruits (Tables 2, 3, Supplementary Material). Moreover, a high amount of ascorbic acid was observed at time 1 with respect to time 2 (1730 and 1016 mg kg^−1^), whereas both quercetin and antioxidant activity were higher at time 2 than time 1 (1.43 and 0.07 μg g^−1^ and 8803 and 2398 mg Fe^2+^ kg^−1^, respectively) (*P* ≤ 0.05) (Tables 2, 3, Supplementary Material). Importantly, at time 2, AH-treated green fruits differed from both RG and UNT for the high content in chlorogenic acid (28.5 vs. 14 and 12 μg g^−1^, respectively) (*P* ≤ 0.05), *p*-hydroxybenzoic acid (1.34 vs. 1.05 and 1.04 μg g^−1^) (*P* ≤ 0.05), *p*-coumaric acid (0.94 vs. 0.32 and 0.37 μg g^−1^) (*P* ≤ 0.05) and antioxidant activity (14819 vs. 13132 and 12071 mg Fe^2+^ kg^−1^) (*P* ≤ 0.05) (Tables 2, 3, Supplementary Material). However, both AH- and RG-treated red fruits at time 2 had a higher amount of capsaicin than UNT2 (277, 291 vs. 48 μg g^−1^, respectively) (*P* ≤ 0.05) (Tables 2, 3, Supplementary Material).

**Table 3:** **^1^H and ^13^C chemical shifts of assigned metabolites**.

**Metabolite**	**Assignment**	**^1^H δ (ppm)**	**Multiplicity [*J*(Hz)]**	**^13^C δ (ppm)**
**CARBOHYDRATES**				
β-glucose	CH-1	4.66	d [7.92]	
	CH-2	3.25	dd	
	CH-3	3.50		
	CH-4	3.41		
	CH-5	3.46		
	CH_2_−6,6′	3.88; 3.72		
α-glucose	CH-1	5.22	d [3.82]	
	CH-2	3.53		
	CH-3	3.72		
	CH-4	3.42		
	CH-5	3.84		
	CH_2_−6,6′	3.83; 3.84		
Sucrose	CH-1 (Glc)	5.42	d [3.90]	93.00
	CH-2 (Glc)	3.57	dd [3.90; 9.98]	71.63
	CH-3 (Glc)		t	72.97
	CH-4	3.48	t [9.39]	69.96
	CH-5	3.86	dd	73.12
	CH2-6	3.81		60.87
	CH2-1′ (Fru)	3.68		61.24
	CH-2′			104.58
	CH-3′	4.22	d [8.80]	77.38
	CH-4′	4.11	t	75.34
	CH-5′	3.88		81.84
	CH2-6′	3.83		62.91
**ORGANIC ACIDS**				
Citric acid	α,γ-CH	2.57	dd [HMQC]	45.82
	α′,γ′-CH	2.69	dd [HMQC]	45.82
Ascorbic acid	CH-4	4.54	d [2.1]	
	CH-5	4.01		
	CH_2_−6	3.73		
Malic acid	α-CH	4.31	dd [10.56; 2.93]	
	ß-CH	2.68	dd [10.56; 2.93]	43.30
	ß′-CH	2.38	dd [15.26; 10.56]	43.30
Acetic acid	CH_3_	1.92	s	
**AMINO ACID**				
Alanine (Ala)	α-CH	3.77		51.28
Arginine (Arg)	α-CH	3.77		54.75
	ß-CH_2_	1.92		27.70
	γ-CH_2_	1.68		24.20
Asparagine (Asn)	α-CH	4.01	dd	50.70 (?)
	ß-CH	2.83	dd [13.94; 8.22]	34.8
	ß′-CH	2.99	dd [13.94; 4.4]	34.8
(GABA)	α-CH_2_	2.30	t [7.30]	34.60
	ß-CH_2_	1.91	m	24.00
	γ-CH_2_	3.02	t [7.5]	39.60
Glutamate (Glu)	α-CH	3.77		55.12
	ß-CH	2.05	m	26.58
	ß'-CH	2.10		26.58
Glutamine (Gln)	α-CH	3.77		54.78
	ß,ß'-CH_2_	2.15	m	26.20
Isoleucine (Ile)	α-CH	3.63		60.78
	ß-CH	1.98		37.78
	γ-CH	1.26		
	γ'-CH	1.48		
	γ-CH_3_	1.02	d [7.00]	16.55
Leucine (Leu)	α-CH	3.74		
	ß-CH_2_	1.75		
	γ-CH	1.68		
	δ-CH_3_	0.96	d [6.00]	
Lysine (Lys)	α-CH	3.77		
	ß-CH_2_	1.91		
	γ-CH_2_	1.49		
	δ-CH_2_	1.72		
Phenylalanine (Phe)	C2, 6, ring	7.40		129.76
	C3, 5, ring	7.36		129.76
Proline (Pro)	α-CH	4.14		
	ß-CH	2.36		31.78
	ß'-CH	2.08		
	γ-CH_2_	2.01		26.59
	δ-CH	3.41		48.84
Threonine (Thr)	α-CH	3.60		
	ß-CH	4.26	m	65.93
Tryptophan (Trp)	CH-4, ring	7.74	d [7.99]	
	CH-5, ring	7.20		
	CH-6, ring	7.29		
	CH-7, ring	7.55	d [8.22]	
Tyrosine (Tyr)	CH-2,6, ring	7.20	d [8.60]	129.38
	CH-3,5, ring	6.91	d [8.60]	117.75
Valine (Val)	α-CH	3.62		
	ß-CH	2.27	m	29.50
	γ-CH_3_	1.00	d [7.04]	16.87
	γ'-CH_3_	1.04	d [7.04]	18.04
**OTHER COMPOUNDS**				
Cytidine	N-CH	7.84	d [7.65]	
	NCH=C*H*	6.05	d [7.55]	
	N-*CH*-O	5.89	d [4.12]	
NADP^+^		9.29	s	
		9.10		
		8.82		
Imidazolederivatives		8.34		
Trigonelline		9.07		
2-oxoglutarate	CH_2_-C=0	2.41		
dCTP/dTTP		6.35		
Thymidine		6.39		
Nicotinate	N-CH	8.59		
1-methylnicotinamide		8.93		

### ^1^H-HRMAS-NMR of red peppers

**Figure 5:**
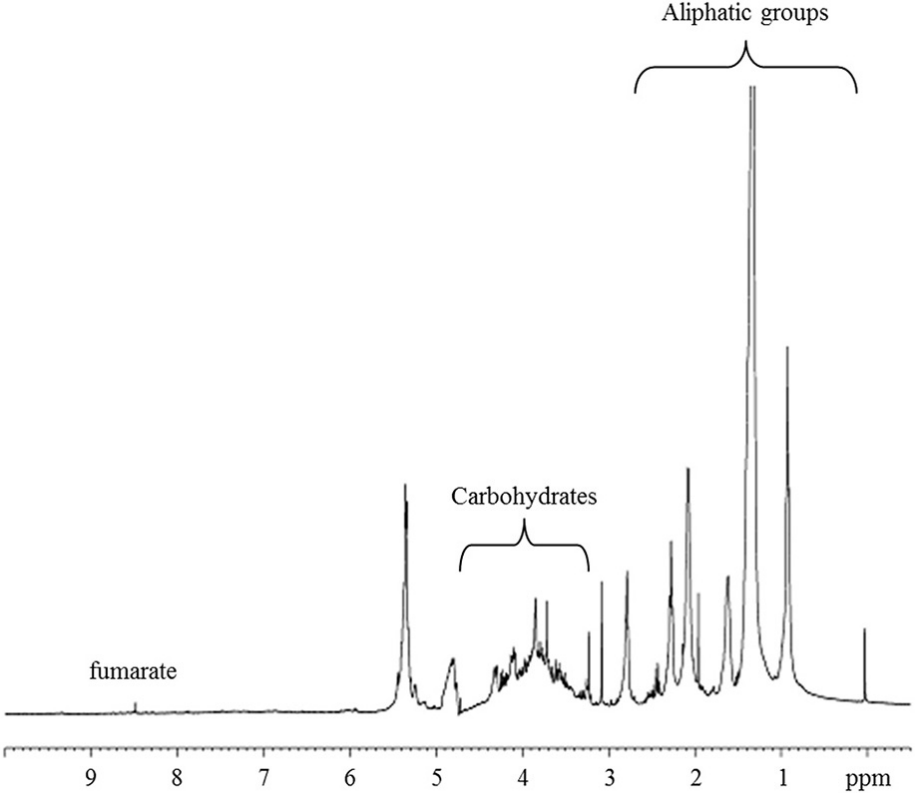
**^1^H-HRMAS-NMR spectrum of red fruits treated with alfalfa hydrolyzed (AH)**.

The metabolite profiles in the ^1^H-HRMAS-NMR spectrum of AH-treated red fruits is shown in Figure 5. The assignments of ^1^H and ^13^C chemical shifts based on 1D- and 2D TOCSY experiments are supported by chemical shifts previously reported in the literature (Ritota et al., 2010), which enabled the identification of different metabolites (Table 3). The spectra were characterized by a broad region in the range 3.5–4.5 ppm, typically attributed to carbohydrates. Furthermore, the anomeric carbon protons of both β-D- and *α*-D-glucose were found at 4.66 and 5.22 ppm, respectively. The signal at 4.66 ppm showed cross-peaks in the TOCSY spectrum with a resonance at 3.25, 3.48, 3.40, 3.43, 3.89, and 3.79 ppm, which is the typical spin system of the glucose moiety. Similarly, for α-D-glucose, we observed in TOCSY correlations between the signals at 5.24, 3.54, 3.71, 3.42, 3.83, and 3.84 (Table 3). Signals from organic acids are assigned to doublets at δ = 2.6 ppm (*J* = 13.9 and 3.1 Hz) due to malate based on the cross-peaks in the TOCSY spectrum with peaks at δ = 2.39 and 4.31 ppm, β-CH and *α*-CH, respectively (Figure 5). The singlet at 6.50 ppm was assigned to fumarate, and the other singlet at 8.46 ppm to formate. Valine, isoleucine, leucine, threonine, alanine, asparagine, arginine, lysine, glutamate, glutamine, *γ*-aminobutyric acid (GABA), phenylalanine, tryptophan, and tyrosine were identified, as previously described in detail by Ritota et al. (2010). In addition, peaks between 7.44 and 7.48 ppm with low intensities were assigned to high-molecular-weight species (HMW) and the resonance at δ = 9.12 ppm showed TOCSY cross-peaks with protons at δ = 8.83 and 8.08 ppm. These were assigned to trigonelline (Ritota et al., 2013).

The PLS-DA classification model (data not shown) was obtained to discriminate the effect of both biostimulants on quantities of the corresponding metabolite in pepper. For example, untreated pepper contained higher amounts of methylnicotinamide, cytidine, trigonelline, and imidazole derivatives compared to pepper treated with AH. Thus, the treated pepper showed the highest amount of NADP^+^. This differed from the effect of RG treatment. This material induced a higher amount of several metabolites, such as glucose, fumarate, ascorbate, thymidine, NADP^+^, nicotinate, deoxycytidine triphosphate (dCTP) and/or deoxythymidine triphosphate (dTTP) and HMW species, while the untreated pepper was characterized by a higher amount in glutamate and/or glutamine and/or 2-oxoglutarate.

## Discussion

Application of AH and RG biostimulants in pepper plant cultivation has demonstrated strong positive effects on growth, development and fruit quality. The beneficial effects observed indicate the presence of more than one group of plant growth-promoting substances/hormones. Interestingly, IAA and IPA were found in both biostimulants at different amounts, as shown in Table 1. Moreover, Infrared and Raman spectra showed a typical spectroscopic pattern of amino acid functional groups in peptidic structure (Schiavon et al., 2008; Ertani et al., 2009) for AH and polyphenols such as resveratrol for RG, which is commonly present in grape skins (Felice et al., 2012). In the case of AH, the presence of a high amount of amino acids and peptides may be due to the proteolytic processes used to obtain the product. These peptides may act as growth factors, which regulate the structure and function of plant tissues and organs (Matsubayashi and Sakagami, 2006). Similarly, these phenolic substances may show hormone-like activity (Pizzeghello et al., 2006) and stimulate the phenylpropanoid pathway (Ertani et al., 2011) similar to HSs, which exert an auxin-mediate signal transduction (Schiavon et al., 2010). Typically, these compounds act independently via classic plant hormones, although a synergistic effect cannot be excluded.

After the first application of AH and RG, the biostimulant effect was visible early in terms of quantitative characteristics, such as a sharp increase in the weight and number of fresh leaves and fruits (i.e., the green fruits). This was a typical short-time effect that has been previously observed in treated plants with HSs or other biostimulants (Nardi et al., 2009; Azcona et al., 2011; Ertani et al., 2012). In a long period, when the plant reached maturity, the main effects were quantified in terms of considerable increases in the total fresh fruit weight as well as the total number of fruits with respect to the control. In particular, red fruits were most affected by the treatments. On the basis of this finding, we can infer that the positive effect of AH and RG was maintained during the entire growth period.

From a metabolic perspective, biostimulants (i.e., HSs) may modulate carbon and nitrogen metabolism by increasing the activity of enzymes involved in glycolysis, the Krebs cycle and nitrate assimilation (Nardi et al., 2009). Carbohydrates, such as glucose and fructose, are considered to represent the basis of plant metabolism (Winter and Huber, 2000). Carbohydrates not only provide the energy required for various metabolic pathways but also provide carbon skeletons for nitrogen metabolism. In our study, after the application of AH and RG, the content of carbohydrates, such as glucose and fructose sharply increased in the leaves and fruits. These data are consistent with previous reports in which treatment with HSs resulted in an increase in carbohydrate metabolism (Nardi et al., 2000; Muscolo et al., 2005). However, we cannot exclude that the accumulation in carbohydrates might also be related to the growth rate and fruit maturation (Hubbard and Pharr, 1992; Fawole and Opara, 2013).

The NMR spectra of red fruits were consistent with high amounts of these carbohydrates. Consistent with this finding, a high level of NADP^+^ was found after treatment with AH and RG, which may be due to the biosynthetic reactions in the Calvin cycle, to assimilate carbon dioxide, which turn in glucose.

Other metabolic pathways involving secondary metabolites appear to be a consequence of the treatments. The total phenolic acids were strongly enhanced in the leaves after the first biostimulant application, whereas fewer variations were found after the second application. In fruits, AH strongly enhanced the total phenolic acids, particularly in green fruits. Red fruits also demonstrated a lower total phenolic acids content compared to green fruits. It has also been established that the concentration of phenols is high in the early stages and then decreases during fruit maturation (Zhang and Hamauzu, 2003; Oboh et al., 2007). Studies performed on phenolic content during the developing of fruits of other species have shown a similar trend (i.e., Shwartz et al., 2009).

Single phenolic compounds in leaves and fruits showed changes in relationship to the biostimulants doses. Caffeic, *p*-coumaric and *p*-hydroxybenzoic acids sharply increased in leaves after the first treatment with both doses of RG and highest dose of AH. However, a high amount of ferulic acid was only found after the second treatment with both doses of AH. An increase in phenolics in plant tissues may enhance plant resistance to stress conditions. Furthermore, it can provide a source of important antioxidants for human health. For example, caffeic and gallic acids inhibit carcinogenesis (Olthof et al., 2001; Raina et al., 2008). Ferulic acid is known to exert an antimicrobial activity and function as precursors to structural polymers such as lignin (Chen et al., 2006). Moreover, biostimulated plants result in the synthesis of phenylpropanoids compounds via an increased activity and gene expression of the phenylalanine (tyrosine) ammonia-lyase enzyme (Schiavon et al., 2010; Ertani et al., 2011).

The most affected fruits were the green fruits after the second application of AH biostimulant, which exhibited a sharper and higher chlorogenic, *p*-hydroxybenzoic and to a lesser extent *p*-coumaric acids than RG and UNT. Phenolic compounds (gallic, protocatechuic, ferulic, o-coumaric, *p*-coumaric, sinapinic, trans-cinnamic and caffeic acids, quercetin, catechin, rutin, and vanillin) are present in commercial cultivars of chili peppers at different maturation stages (Troconis-Torres et al., 2012). In our study, the abundance of epicatechic, caffeic, and chlorogenic acids was increased in red fruits after the second application of both biostimulants.

Other biologically active compounds such as ascorbic acid, β-carotene and antioxidant activity had sharply higher increases in the treated fruits after the second application of both biostimulants. Ascorbic acid increased 2.5-fold in green fruits treated with AH at low dose, whilst it increased 1.28-fold in red fruits after the second application of RG at the highest dose with respect to untreated fruits. Previous studies found a high level of ascorbic acids in peppers amended with organic wastes (Hallmann and Rembialkowska, 2008; Pascual et al., 2010).

The antioxidant activity was always high with the most optimal increase after the first application in green fruits treated with low doses of AH (1.6-fold UNT). However, red fruits at time 1 and 2 and green fruits at time 2 also exhibited considerable antioxidant activity (1.25–1.46-fold UNT). Importantly, lycopene was found only in fruits at maturity and its amount showed low to high increases in the treated fruits. Capsaicin is an active component, which accounts for the pharmaceutical properties of peppers. It is well established that the level of capsaicin in a given variety can vary depending on the light intensity and temperature at which the plant is grown, the age of the fruit, and the position of the fruit on the plant. In our study, the amount of capsaicin sharply increased *ca* 7-fold in red fruits after the second application of AH and RG at low doses. However, our results differed from those of Pascual et al. (2010), who found that the addition of sewage sludge to soil did not affect the capsaicin and dihydrocapsaicin concentration in pepper fruits.

Most frequently, biostimulants as well as humic substances may modulate nitrogen metabolism (Schiavon et al., 2008; Ertani et al., 2009). For example, in our study, a large amount of methylnicotinamide and consequently of trigonelline was found in untreated pepper fruits compared to plants treated with AH. These metabolites may function as signal transmitters in the response of plants to oxidative stress (Berglund, 1994). Furthermore, it has been suggested that the physiological effects of trigonelline and other quaternary ammonium compounds in plants could occur at the level of DNA methylation (Kraska and Schönbeck, 1993).

## Conclusion

On the basis of our data, the two biostimulants demonstrated ameliorating properties on the growth of pepper plants from flowering to fruit maturity. With this perspective, long-term experiments should collect information on the nutritive and health-promoting compounds of a biostimulated crop.

The use of biostimulants should also be increased to reduce the excessive inputs of mineral fertilization, which is a source of pollution and a risk for environmental fitness. Moreover, from a management perspective, cultivation wastes might turn into raw materials, thus permitting a reduction in disposal costs.

### Conflict of interest statement

The authors declare that the research was conducted in the absence of any commercial or financial relationships that could be construed as a potential conflict of interest.
