# Supplementary material for: Capsicum chinensis L. growth and nutraceutical properties are enhanced by biostimulants in a long-term period: chemical and metabolomic approaches
Source: Front Plant Sci. 2014 Aug 1;5:375. doi: 10.3389/fpls.2014.00375 (PMC4117981; doi:10.3389/fpls.2014.00375)
Supplement: Supplementary file 1 [file DataSheet1.DOCX]

**Supplementary Material**

Supplementary Table 1. Leaves chemical composition after the 1^st^ and 2^nd^ (T1 and T2) application of biostimulants (untreated, UNT; red grape, RG and alfalfa hydrolysed, AH).

| T | Tr | C |  | Glu | Fru |  | TP |  | Chl | Caf | Fer | Coum | Hyd | Epi | Que | β-car |  | AA | As |
| --- | --- | --- | --- | --- | --- | --- | --- | --- | --- | --- | --- | --- | --- | --- | --- | --- | --- | --- | --- |
|  | mL L^-1^ | |  | mg g^-1^ dw | |  | mg GAE kg^-1^ fw |  | µg g^-1^ dw | | | | | | | |  | mg Fe^2+^ kg^-1^ fw | mg kg^-1^ fw |
| 1 | UNT | 0 |  | 247c | 100d |  | 1106c |  | nd | 1.58b | 1.34c | 0.45d | 0.88c | 6.49c | 0.51b | 1.84b |  | 3047c | 1861c |
| 1 | RG | 50 |  | 240c | 188c |  | 1166c |  | nd | 3.83a | 0.83d | 1.60a | 1.17b | 9.49a | 0.31ab | 2.13ab |  | 2907c | 1712d |
| 1 | RG | 100 |  | 452a | 232a |  | 1368b |  | nd | 3.72a | 2.79b | 1.46b | 1.10b | 9.66a | 0.61a | 2.35a |  | 3211b | 1968b |
| 1 | AH | 25 |  | 370b | 214b |  | 1693a |  | nd | 1.51b | 1.21c | 0.73d | 0.26d | 7.39b | 0.33c | 1.86b |  | 3696a | 2192a |
| 1 | AH | 50 |  | 436a | 225a |  | 1750a |  | nd | 3.58a | 5.45a | 1.75a | 1.38a | 7.66b | 0.69a | 2.34a |  | 3782a | 1908b |
|  |  |  |  |  |  |  |  |  |  |  |  |  |  |  |  |  |  |  |  |
| 2 | UNT | 0 |  | 719*a* | 352*b* |  | 1317*c* |  | 53*b* | 5.05*c* | 6.32*b* | 4.16*a* | 1.05*a* | 1.39*c* | nd | 1.58*c* |  | 3623*b* | 528*c* |
| 2 | RG | 50 |  | 489*c* | 255*d* |  | 1785*b* |  | 40*c* | 2.89*d* | 3.53*c* | 1.61*d* | 0.84*b* | 1.56*c* | nd | 1.77*b* |  | 3747*b* | 618*b* |
| 2 | RG | 100 |  | 691*a* | 324*c* |  | 1647*b* |  | 48*b* | 9.33*b* | 4.34*c* | 3.85*b* | 0.91*a* | 2.75*a* | nd | 1.76*b* |  | 3635*b* | 652*a* |
| 2 | AH | 25 |  | 627*b* | 386*a* |  | 1907*a* |  | 51*b* | 15.22*a* | 11.21*a* | 1.39*d* | 0.06*c* | 2.28*b* | nd | 1.88*a* |  | 3892*a* | 723*a* |
| 2 | AH | 50 |  | 531*c* | 288*d* |  | 1896*a* |  | 81*a* | 6.05*c* | 12.20*a* | 2.07*e* | 0.05*c* | 2.13*b* | nd | 1.66*bc* |  | 3992*a* | 520*c* |

T, time; Tr, treatment; C, concentration. AA, antioxidant activity; As, ascorbic acid; β-car, β-carotene; Caf, caffeic acid; Chl, chlorogenic acid; Coum, *p*-coumaric acid; Epi, epicatechic acid; Fer, ferulic acid; Fru, fructose; Glu, glucose; Hyd, *p*-hydroxybenzoic acid; Que, quercetin; TP, total phenols. In the same column differences among means at time 1 and at time 2 (italicised letters) were at P≤0.05. Supplementary Table 2. Chemical composition of green (G) and red (R) fruits after the 1^st^ application (Time 1) of biostimulants (untreated, UNT; red grape, RG and alfalfa hydrolysed, AH).

|  | Tr | C |  | Glu | Fru |  | TP |  | Chl | Caf | Fer | Coum | Cin | Hyd | Epi | Que | β-car | Cap | Dih |  | AA | As |
| --- | --- | --- | --- | --- | --- | --- | --- | --- | --- | --- | --- | --- | --- | --- | --- | --- | --- | --- | --- | --- | --- | --- |
|  |  | mL L^-1^ |  | mg g^-1^ dw | |  | mg GAE kg^-1^ fw |  | µg g^-1^ dw | | | | | | | | | | |  | mg Fe^2+^ kg^-1^ fw | mg kg^-1^ fw |
| G | UNT | 0 |  | 883a | 436c |  | 3623b |  | 23.1b | 0.48c | 0.49c | 0.62a | 0.10c | 0.99c | 4.09a | 0.08c | nd | 181a | 46a |  | 1074d | 1497c |
|  | RG | 50 |  | 789b | 594a |  | 2789c |  | 9.4d | 0.25e | 0.72b | 0.60a | 0.14c | 1.00c | 0.94c | 0.11b | nd | 184a | 40b |  | 944e | 1695b |
|  | RG | 100 |  | 529d | 441c |  | 5548a |  | 18.7c | 0.33d | 1.09a | 0.40b | 0.17a | 1.24a | 3.05b | 0.23a | nd | 152b | 32c |  | 1581b | 1637b |
|  | AH | 25 |  | 873a | 553b |  | 5545a |  | 16.1c | 0.59b | 1.14a | 0.37b | 0.15b | 1.08b | 3.30b | 0.01d | nd | 154b | 38b |  | 1743a | 1810a |
|  | AH | 50 |  | 647c | 438c |  | 5467a |  | 31.5a | 0.82a | 0.69b | 0.39b | 0.15b | 1.13b | 4.20a | 0.01d | nd | 164b | 47a |  | 1434c | 1699b |
|  |  |  |  |  |  |  |  |  |  |  |  |  |  |  |  |  |  |  |  |  |  |  |
| R | UNT | 0 |  | 2637*b* | 2381*b* |  | 1086*d* |  | 0.90*c* | 0.36*b* | 1.96*a* | 0.46*b* | 0.24*b* | 0.74*c* | 1.45*a* | 0.05 | 1.26*b* | 157*b* | 44*b* |  | 3091*c* | 1741*c* |
|  | RG | 50 |  | 1546*e* | 1690*e* |  | 1080*d* |  | 1.21*b* | 0.29*c* | 0.83*b* | 0.18*e* | 0.05*d* | 0.49*d* | 0.23*d* | 0.04 | 1.23*c* | 193*a* | 35*c* |  | 3178*b* | 1677*d* |
|  | RG | 100 |  | 1830*c* | 2004*c* |  | 1677*a* |  | 0.91*c* | 0.25*c* | 2.05*a* | 0.36*d* | 0.26*b* | 0.85*b* | 1.43*a* | 0.06 | 1.30*a* | 122*c* | 26*d* |  | 4511*a* | 1751*c* |
|  | AH | 25 |  | 2763*a* | 3005*a* |  | 1313*b* |  | 2.62*a* | 0.52*a* | 2.06*a* | 0.41*c* | 0.15*c* | 0.93*a* | 0.32*c* | 0.04 | 1.25*b* | 203*a* | 39*c* |  | 3188*b* | 1913*a* |
|  | AH | 50 |  | 1741*d* | 1882*d* |  | 1137*c* |  | 2.74*a* | 0.39*b* | 2.01*a* | 0.56*a* | 0.40*a* | 1.12*a* | 1.18*b* | 0.07 | 1.32*a* | 209*a* | 63*a* |  | 3235*b* | 1878*b* |

Tr, treatment; C, concentration. AA, antioxidant activity; As, ascorbic acid; β-car, β-carotene; Caf, caffeic acid; Cap, capsaicin; Chl, chlorogenic acid; Cin, cinnamic acid; Coum, *p*-coumaric acid; Dih, dihydrocapsaicin; Epi, epicatechic acid; Fer, ferulic acid; Fru, fructose; Glu, glucose; Hyd, *p*-hydroxybenzoic acid; Que, quercetin; TP, total phenols. In the same column differences among means for G and R fruits (italicised letters) were at P≤0.05.

Supplementary Table 3. Chemical composition of green (G) and red (R) fruits after the 2^nd^ application (Time 2) of biostimulants (untreated, UNT; red grape, RG and alfalfa hydrolysed, AH).

|  | Tr | C |  | Glu | Fru |  | TP |  | Chl | Caf | Fer | Coum | Cin | Hyd | Epi | Que | β-car | Lyc | Cap | Dih |  | AA | As |
| --- | --- | --- | --- | --- | --- | --- | --- | --- | --- | --- | --- | --- | --- | --- | --- | --- | --- | --- | --- | --- | --- | --- | --- |
|  | mL L^-1^ | |  | mg g^-1^ dw | |  | mg GAE kg^-1^ fw |  | µg g^-1^ dw | | | | | | | | | | | |  | mg Fe^2+^ kg^-1^ fw | mg kg^-1^ fw |
| G | UNT | 0 |  | 211d | 223d |  | 2351c |  | 12d | 0.32e | 1.70b | 0.37b | 0.22c | 1.04b | 0.07c | 1.42b | 1.48c | 0.03c | 89c | 27c |  | 12071e | 497d |
|  | RG | 50 |  | 884b | 387d |  | 3435b |  | 18c | 0.73b | 1.80b | 0.25c | 0.23c | 1.05b | 0.10c | 1.78a | 1.53b | 0.05b | 167a | 29c |  | 12560d | 705c |
|  | RG | 100 |  | 1603a | 733a |  | 5214a |  | 10d | 0.66c | 1.74b | 0.40b | 0.32a | 1.10b | 0.12b | 1.39b | 1.68a | 0.08° | 160a | 54a |  | 13704c | 1029b |
|  | AH | 25 |  | 866b | 644b |  | 5648a |  | 25b | 0.41d | 3.16a | 0.96a | 0.27b | 1.37a | 0.16a | 1.44b | 1.54b | 0.04b | 97c | 25c |  | 15162a | 1246a |
|  | AH | 50 |  | 648c | 634b |  | 5673a |  | 32a | 0.82a | 1.90b | 0.91a | 0.29b | 1.31a | 0.08c | 1.45b | 1.57b | 0.05b | 117b | 43b |  | 14476b | 1028b |
|  |  |  |  |  |  |  |  |  |  |  |  |  |  |  |  |  |  |  |  |  |  |  |  |
| R | UNT | 0 |  | 1694*a* | 1730*b* |  | 1195*d* |  | 0.9*d* | 0.21*d* | 1.72*b* | 0.66*a* | 0.46*a* | 1.07*a* | 0.10*c* | 0.91*c* | 1.38*d* | 0.05*b* | 48*d* | 18*d* |  | 4215*c* | 1030*c* |
|  | RG | 50 |  | 1532*d* | 1751*a* |  | 1426*c* |  | 2.5*c* | 0.48*c* | 1.76*b* | 0.34*c* | 0.32*b* | 0.69*c* | 0.41*b* | 0.92*c* | 1.47*c* | 0.06*b* | 319*a* | 70*b* |  | 4627*b* | 1083*c* |
|  | RG | 100 |  | 1558*c* | 1545*d* |  | 1686*b* |  | 1.5*d* | 0.65*b* | 1.07*c* | 0.41*b* | 0.19*c* | 0.53*d* | 0.85*a* | 1.13*c* | 1.44*c* | 0.06*b* | 263*b* | 50*c* |  | 4758*b* | 1322*a* |
|  | AH | 25 |  | 1638*b* | 1685*c* |  | 1737*a* |  | 3.3*b* | 0.93*a* | 1.71*b* | 0.42*b* | 0.18*c* | 0.74*c* | 0.20*c* | 1.78*b* | 1.65*b* | 0.08*a* | 219*c* | 185*a* |  | 5780*a* | 1049*c* |
|  | AH | 50 |  | 1214e | 1353*e* |  | 1671*a* |  | 4.5*a* | 0.98*a* | 2.00*a* | 0.39*b* | 0.19*c* | 0.91*b* | 0.18*c* | 2.09*a* | 1.83*a* | 0.08*a* | 335*a* | 7*b* |  | 676*d* | 1175*b* |

Tr, treatment; C, concentration. AA, antioxidant activity; As, ascorbic acid; β-car, β-carotene; Caf, caffeic acid; Cap, capsaicin; Cin, cinnamic acid; Coum, *p*-coumaric acid; Dih, dihydrocapsaicin; Epi, epicatechic acid; Fer, ferulic acid; Fru, fructose; Glu, glucose; Hyd, *p*-hydroxybenzoic acid; Lyc, lycopene; Que, quercetin; TP, total phenols. In the same column differences among means for G and R fruits (italicised letters) were at P≤0.05.
